# A One-Shot Shift from Explore to Exploit in Monkey Prefrontal Cortex

**DOI:** 10.1523/JNEUROSCI.1338-21.2021

**Published:** 2022-01-12

**Authors:** Jascha Achterberg, Mikiko Kadohisa, Kei Watanabe, Makoto Kusunoki, Mark J. Buckley, John Duncan

**Affiliations:** ^1^MRC Cognition and Brain Sciences Unit, University of Cambridge, CB2 7EF, Cambridge, United Kingdom; ^2^Department of Experimental Psychology, University of Oxford, OX2 6GG, Oxford, United Kingdom; ^3^Graduate School of Frontier Biosciences, 565-0871, Osaka University, Osaka, Japan

**Keywords:** attention, exploit, explore, frontal cortex, one-shot learning, primate

## Abstract

Much animal learning is slow, with cumulative changes in behavior driven by reward prediction errors. When the abstract structure of a problem is known, however, both animals and formal learning models can rapidly attach new items to their roles within this structure, sometimes in a single trial. Frontal cortex is likely to play a key role in this process. To examine information seeking and use in a known problem structure, we trained monkeys in an explore/exploit task, requiring the animal first to test objects for their association with reward, then, once rewarded objects were found, to reselect them on further trials for further rewards. Many cells in the frontal cortex showed an explore/exploit preference aligned with one-shot learning in the monkeys' behavior: the population switched from an explore state to an exploit state after a single trial of learning but partially maintained the explore state if an error indicated that learning had failed. Binary switch from explore to exploit was not explained by continuous changes linked to expectancy or prediction error. Explore/exploit preferences were independent for two stages of the trial: object selection and receipt of feedback. Within an established task structure, frontal activity may control the separate processes of explore and exploit, switching in one trial between the two.

**SIGNIFICANCE STATEMENT** Much animal learning is slow, with cumulative changes in behavior driven by reward prediction errors. When the abstract structure a problem is known, however, both animals and formal learning models can rapidly attach new items to their roles within this structure. To address transitions in neural activity during one-shot learning, we trained monkeys in an explore/exploit task using familiar objects and a highly familiar task structure. When learning was rapid, many frontal neurons showed a binary, one-shot switch between explore and exploit. Within an established task structure, frontal activity may control the separate operations of exploring alternative objects to establish their current role, then exploiting this knowledge for further reward.

## Introduction

Much animal learning occurs slowly, with prediction errors leading to incremental changes in the link between actions and their outcomes (Rescorla and Wagner, 1972; Schultz et al., 1997). A similar process of incremental change underlies powerful formal learning models (LeCun et al., 2015; Schmidhuber, 2015). Animals and formal models are also capable, however, of rapid, sometimes one-shot learning. When the abstract structure or schema of a problem is known, new items can rapidly be attached to their roles within this structure (“variable binding”) (Smolensky, 1990). Familiar examples include learning to learn (Harlow, 1949), object-location binding (Behrens et al., 2018), and meta-learning (Wang et al., 2018). One-shot variable binding is conspicuous throughout human cognition, endowing thought and behavior with their characteristic speed, flexibility, and compositionality (Lake et al., 2017).

Frontal cortex contributes to rapid learning. In a block of trials, frontal population activity shows abrupt changes when new task rules are adopted (Durstewitz et al., 2010; Emberly and Seamans, 2020) or object-reward bindings must be reversed (Bartolo and Averbeck, 2020). Frontal neurons are well known to encode trial-unique rules, or items to be maintained in working memory (Fuster et al., 1982; Miller et al., 1996; Wallis et al., 2001; Mansouri et al., 2006). Reflecting rapid learning on each trial, this information indicates how individual decisions should be taken within a well-learned task structure.

In pioneering studies, Procyk and colleagues (Procyk and Goldman-Rakic, 2006; Quilodran et al., 2008; Rothe et al., 2011; Khamassi et al., 2015; Enel et al., 2016) examined one-trial transition from unknown to known task rules in a spatial selection task. In this task, monkeys selected different screen locations in turn, searching for the one location associated with reward (“explore” trials). Once reward was found, the same location could be selected on a series of further trials (“exploit” trials) for further rewards. Monkeys performed this task close to perfectly, with immediate transition from explore to exploit once the rewarded location was found. At this transition, spatial selectivity declined in neurons of dorsolateral frontal cortex (Procyk and Goldman-Rakic, 2006; but see Khamassi et al., 2015), and response to feedback decreased in anterior cingulate neurons (Quilodran et al., 2008).

To extend these findings, and to separate learning from motor planning, we designed a similar comparison of explore and exploit trials in an object selection task. We examined explore/exploit preferences during two stages of each trial, choice and feedback. We focused on activity in lateral PFC, with comparison data from inferior parietal cortex. A previous report of data from this task focuses on the dynamics of object and location selectivity within each trial (Kadohisa et al., 2020). Here, separate from object and location coding, we examine the transition from a frontal explore to exploit state, including one-shot switches with successful learning, and maintenance of the explore state when learning fails.

Human brain imaging suggests that first encounters with a new problem lead to strong activity in lateral frontal cortex and other cognitive control regions, which rapidly decreases once the solution is found (Konishi et al., 1998; Hampshire and Owen, 2006), sometimes accompanied by increasing activity in other brain regions, including the basal ganglia (Ruge and Wolfensteller, 2013). These results suggest rapid transfer of control from frontal cortex to other regions with task repetition. In contrast to this, we show bidirectional activity changes in the frontal cell population, with some cells selectively activating for explore, shifting in one trial to others selectively activating during exploit. These different activity patterns, we propose, may contribute to the different computations underlying learning and use of task rules.

## Materials and Methods

### Subjects and procedure

**Figure 1.**
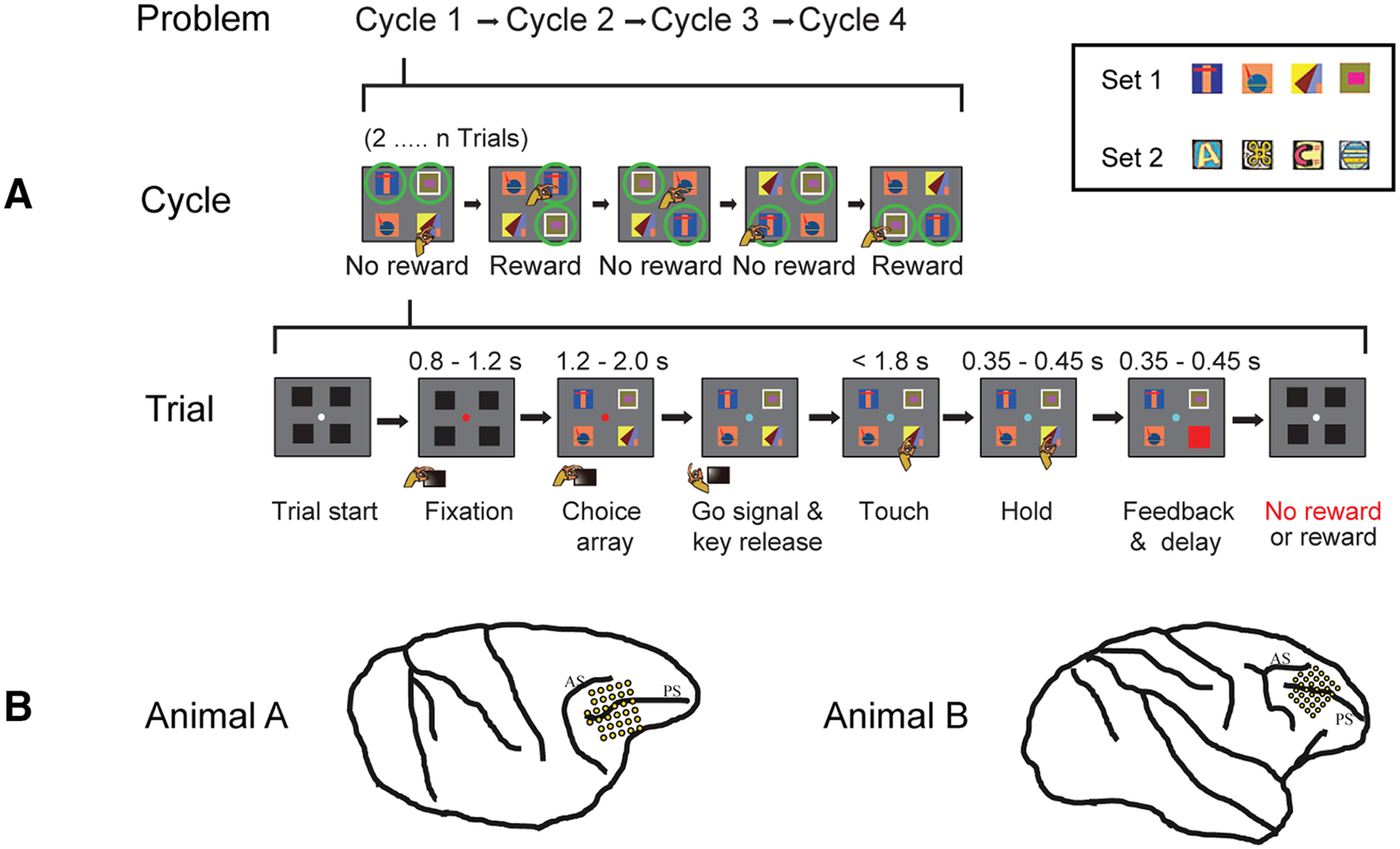
Task overview and recording locations. ***A***, Object selection task, 2-target version. In each session, the animal worked through a series of problems, each consisting of four cycles of trials (top row). On each trial (bottom row), the monkey touched a single object in a visual display. For each problem, two objects were defined as targets; the monkey was rewarded for selecting each target once per cycle, with the cycle ending as soon as both targets had been selected. For each new problem, in the first set of trials (Cycle 1), the monkey selected one object after another, learning which two objects (targets) were associated with reward. An example cycle consisting of five trials is illustrated in the middle row. In this row, icons for each trial indicate stimulus display, animal's choice, and delivery of reward or no reward. Green circles represent targets (not present on actual display). Within a cycle, revisiting a target already selected did not bring further reward (see fourth trial in example). In each subsequent cycle (Cycles 2-4), the animal could reselect the same targets for further rewards, again avoiding revisits within a cycle. Thus, Cycle 1 consisted of a series of 2…*n* trials, continued until the two targets had been found. Cycles 2-4 optimally consisted of just two trials each, one for each target. After four cycles, targets were redefined for the next problem. Alternate problems used two different 4-object sets, fixed for each animal (object sets for one animal in inset). Equivalent 1-target problems (not illustrated) had only a single target; the first cycle ended as soon as this single target was found; and optimally, each subsequent cycle consisted of just a single trial on which this same target was reselected. ***B***, Recording areas in each animal. PS, Principal sulcus; AS, arcuate sulcus.

Data were recorded from 2 male rhesus monkeys, across a total of 60 daily sessions. Before recordings began, animals were trained in increasingly complex task versions, with several months of training in the final version until proficiency was sufficient to provide stable neurophysiological data. Recordings used a semi-chronic microdrive system (SC-32, Gray Matter Research, 1.5 mm interelectrode spacing), with one 32-channel array over lateral frontal cortex (Fig. 1*B*), the primary focus of the current report (Monkey A: AP = 33.9, ML = 20.3; Monkey B: AP = 36.2, ML = 58.1), and another over parietal cortex (Monkey A: AP = −4.6, ML = 50.6; Monkey B: AP = −3.2, ML = 47.4). We did not preselect neurons for task-related responses; instead, we advanced microelectrodes until we could isolate neuronal activity before starting the task. The microdrive system interfaced to a multichannel data acquisition system (Cerebus System, Blackrock Microsystems). Between recording sessions, electrodes were advance by a minimum of 62.5 μm to ensure recordings of new cells. We amplified and filtered (300 Hz to 10 kHz) the neural activity before using it for offline cluster separation and analysis (Offline Sorter, Plexon). Eye position was sampled at 120 Hz using an infrared eye tracking system (Applied Science Laboratories) and stored for offline analysis. All analyses were conducted with the Anaconda Python Distribution (Python Software Foundation, Anaconda) using the packages NumPy (Harris et al., 2020), pandas (McKinney, 2010), SciPy (Virtanen et al., 2020), Matplotlib (Hunter, 2007), seaborn (Waskom, 2021), Pingouin (Vallat, 2018), statsmodels (Seabold and Perktold, 2010), IPython (Perez and Granger, 2007), and Jupyter (Kluyver et al., 2016).

At the end of the experiments, animals were deeply anesthetized with barbiturate and then perfused through the heart with heparinized saline followed by 10% formaldehyde in saline. The brains were removed for histology and recording locations confirmed.

All surgeries were aseptic and conducted under general anesthesia. The experiments were performed in accordance with the Animals (Scientific Procedures) Act 1986 of the United Kingdom; all procedures were licensed by a Home Office Project License obtained after review by Oxford University's Animal Care and Ethical Review committee, and were in compliance with the guidelines of the European Community for the care and use of laboratory animals (EUVD, European Union directive 86/609/EEC).

### Task

In each session, the animal worked through a series of problems, each consisting of a series of trials organized into four cycles (Fig. 1*A*, top). Each problem was based on a set of four objects, with the choice display for each trial containing all four of these, randomly positioned (Fig. 1*A*). For each new problem, one or two objects were randomly defined as targets, bringing reward when touched. In a first cycle of trials (“explore”), the monkey learned by trial and error which objects were targets. In later cycles (“exploit”), targets could be reselected for further rewards. Each problem, accordingly, required new object-role bindings to be learned. For each animal, there were two sets of four objects, fixed throughout the experiment, and used in alternate problems in each session.

In the first cycle (Fig. 1*A*, middle row), the monkey sampled objects in turn across a series of trials, searching for the rewarded target or targets. 1-target and 2-target problems were blocked, so the animal knew in advance how many to discover (mean of 69 1-target and 67 2-target problems per session). Once targets were found, there followed three exploit cycles, in which animals were rewarded for reselecting the targets discovered in Cycle 1. In 2-target problems, the animal was free to select the two targets in each cycle in either order, but revisiting a target already selected in this cycle was not rewarded again. All cycles ended as soon as the single target (1-target problems) or two targets (2-target problems) had been selected. Optimally, therefore, the explore cycle consisted of a random sequence of object selections, avoiding revisits, until the single target (mean expected number of trials = 2.50) or two targets (mean expected number of trials = 3.33) were discovered. Exploit cycles consisted optimally of just one (1-target problems) or two (2-target problems) trials.

Details of events on each trial are illustrated in Figure 1*A* (bottom). Before the trial began, the screen showed a central white fixation point (FP) and a surrounding display of 4 black squares (each square 5.7 × 5.7 deg visual angle, centered 11.4 deg from fixation). To initiate trial events, the monkey was required to press and hold down the start key, and to acquire and hold central fixation (window 7.6 × 7.6 deg). At this point, the FP turned red, and there was a wait period of 0.8-1.2 s, after which the black squares were replaced by a display (choice array [CH]) of four choice objects. Following a further delay of 1.2-2.0 s, the FP changed to cyan (GO) to indicate that a response could be made. To indicate his choice, the animal released the start key and touched one of the objects (touch required within 1.8 s of GO). After the touch had been held for 0.35-0.45 s, the selected object was replaced by either a green (correct target touch) or red (incorrect) square (feedback, FB), which remained for 0.3 s followed by an intertrial display. If the touch was correct, a drop of soft food was delivered 0.05-0.15 s after FB offset. Once a trial had been initiated, it was aborted without reward if the monkey released the start key or broke fixation before GO. The trial was also aborted if, after an object had been touched, the touch was not maintained until FB.

Different intertrial displays indicated transitions within a cycle, between cycles, and between problems. For trials within a cycle, the intertrial display was simply the white FP and surrounding black squares (Fig. 1*A*), with a minimum period of 0.7-0.9 s required before the next trial would begin. To indicate the end of a cycle, this display was preceded by a period of only the white FP, lasting 3.2-3.5 s. To indicate the end of a problem, the screen blanked for 3.3-3.6 s.

### Data analysis

To produce peristimulus time histograms (PSTHs) (see Figs. 3, 5, 7, 8), we counted spikes in 100 ms windows, starting at a window centered at −200 ms from CH or FB and then shifting in 25 ms steps to a final window centered at 475 ms. Spike counts in each window were divided by an estimate of the cell's mean activity, defined as mean activity across all conditions in the CH and FB ANOVAs used for cell selection (see Results). To create the PSTH for each cell, within each time window, we calculated unweighted mean activity across number of targets (1, 2) × object set (1, 2) × touched location (1-4).

For all analyses, we excluded problems in which animals failed to respond on 6 or more trials in a single cycle, suggesting poor task focus.

## Results

### Behavior

**Figure 2.**
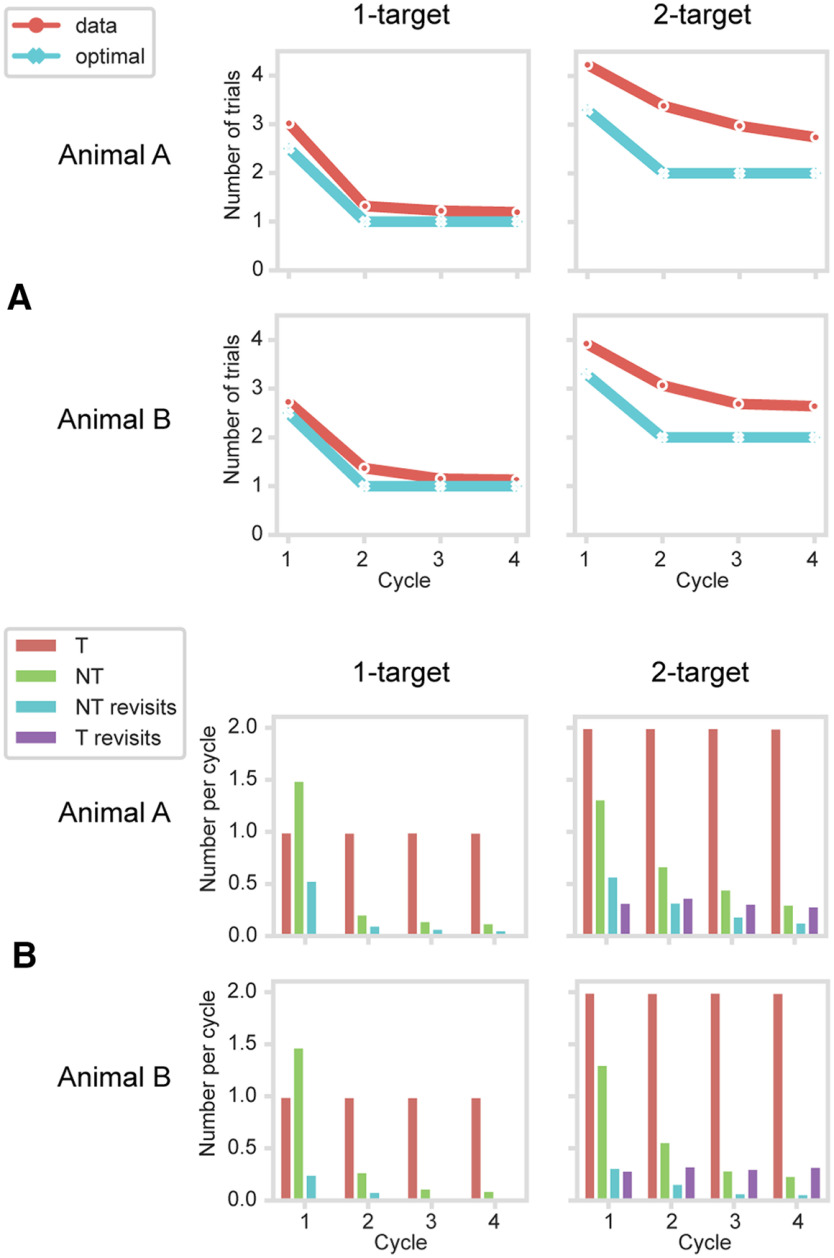
Behavioral data. Split by animal (rows) and type of problem (columns). ***A***, Mean number of trials per cycle. ***B***, Trials per cycle broken down into correct target selections (T), novel nontarget selections (selection of a nontarget not previously sampled in this cycle [NT]), repeat nontarget selections (selection of a nontarget previously sampled in this cycle [NT revisits]), and repeat target selections (T revisits; only possible for 2-target problems).

Behavioral data for each animal are summarized in Figure 2, separately for 1-target (left column) and 2-target (right column) problems. As noted above, for the explore cycle, optimal performance consisted of a random sequence of object selections, avoiding revisits, until the single target (mean expected number of trials = 2.50) or two targets (mean expected number of trials = 3.33) were discovered. Exploit cycles consisted optimally of just one (1-target problems) or two (2-target problems) trials. In 1-target problems, the mean number of trials per cycle was close to optimal (Fig. 2*A*, left; data in red, optimal possible performance in blue), indicating rapid, generally one-trial learning. In 2-target problems (Fig. 2*A*, right), performance improved more gradually over cycles, showing slower learning. A more detailed breakdown of response types is shown in Figure 2*B*. In each cycle, the number of correct target selections (red) was by definition one (1-target problems) or two (2-target problems). As expected, novel nontarget selections (selection of a nontarget not previously sampled in this cycle) were frequent in Cycle 1, occurring in the proportions required by a random search. Rapid discrimination between targets and nontargets is shown by the substantial decline in nontarget selections across cycles, clearly evident in both 1-target and 2-target problems. For each animal, and for each problem type, ANOVA entering separate mean data for each session showed a significant decline in nontarget selections across cycles (all *F* > 22, *p* < 0.0001). For 1-target problems, Tukey HSD tests showed significant differences between Cycles 1 and 2 (*p* < 0.001 for each animal), between Cycles 2 and 3 for just one animal (*p* = 0.64 and *p* = 0.05, respectively, for Animals A and B), and no difference between Cycles 3 and 4 (both *p* > 0.60). For 2-target problems, there were significant differences between Cycles 1 and 2 and between Cycles 2 and 3 (all *p* < 0.001), but not between Cycles 3 and 4 (both *p* > 0.3). Revisits to an object already sampled in a cycle were infrequent throughout (Fig. 2*B*: aqua represents nontarget revisits; purple represents target revisits; impossible for 1-target problems).

A further analysis of Cycle 1 data (Table 1) confirms this strong avoidance of objects already sampled. For this analysis, Cycle 1 trials were broken down according to the number of objects already sampled, from 0 (first trial of cycle) to 3. The table shows mean percentage of trials with revisit to a previously sampled object, compared with expected values for a random selection. In all cases, revisit percentage was far below the chance expectation, verified by χ^2^ tests (all χ^2^ > 2000, *p* < 0.0001).

**Table 1.** Successful avoidance of reselection in Cycle 1*^a^*

Already sampled	Animal A		Animal B		Expected
	1-target problems	2-target problems	1-target problems	2-target problems	
0	0%	0%	0%	0%	0%
1	6.4%	5.7%	2.9%	6.2%	25%
2	26.1%	24.4%	12.1%	17%	50%
3	55.5%	54.2%	40.6%	44%	75%

^*a*^Separate data for each animal: Percentage of trials with reselection of a previously sampled object, as a function of number of objects already sampled.

Together, these data illustrate a well-established task model, incorporating the monkey's knowledge of abstract task structure. Within this model, there was rapid learning, with strong avoidance of objects already sampled within a cycle, including rewarded targets, and from Cycle 2 onwards, excellent discrimination between targets and nontargets, especially in 1-target problems.

### Prefrontal cells show preference for explore or exploit

Across 60 task sessions, we recorded activity from 254 cells (176 from Monkey A and 78 from Monkey B) in a region spanning the principal sulcus and adjacent dorsolateral and ventrolateral frontal convexities. Except where otherwise specified, data were analyzed just from correct trials (i.e., those on which a current target object was selected).

For our first analysis, we asked whether frontal neurons differentiate the processes of explore, seeking new information to bind into the problem structure versus exploit, using known information to guide behavior. To give the strongest measure of explore/exploit preferences, we focused initially on a comparison of Cycles 1 and 4, combining data from the rapidly learned 1-target problems and the more slowly learned 2-target problems. We analyzed data from two trial phases (Fig. 1*A*, bottom row): choice, the period following onset of the choice array (CH), and feedback, the period following onset of feedback (FB). To ensure unbiased results, we adopted a cross-validated approach. For each cell, trials were randomly assigned to one of two datasets. The first dataset was used for selection of cells (selection dataset), and the second dataset was used to validate the results (validation dataset) as described in the following sections. The selection dataset only contained data for Cycles 1 and 4, as Cycles 2 and 3 were not used for selection. On the selection dataset, we performed ANOVA with factors cycle (1, 4) × number of targets (1, 2) × object set (1,2) × touched location (1-4). These ANOVAs used data from two 400 ms windows, beginning at onset of CH and FB, with a separate ANOVA for each window. For each analysis window, cells with a significant (*p* < 0.05) main effect of cycle were classified as “explore” (spike rate Cycle 1 > 4) or “exploit” (spike rate Cycle 1 < 4). These labels were used simply to distinguish the two groups of cells, with no implications concerning potential functional significance. For each explore or exploit cell, we extracted PSTHs from the validation dataset. For a more complete view of the data, these unbiased PSTHs extended across a longer period (−200 to 500 ms from event onset). PSTHs for each cell were normalized and then averaged across cells within each group (see Materials and Methods). *t* tests across cells, again using 0-400 ms windows, were used to confirm significant cycle preference in the validation dataset.

**Figure 3.**
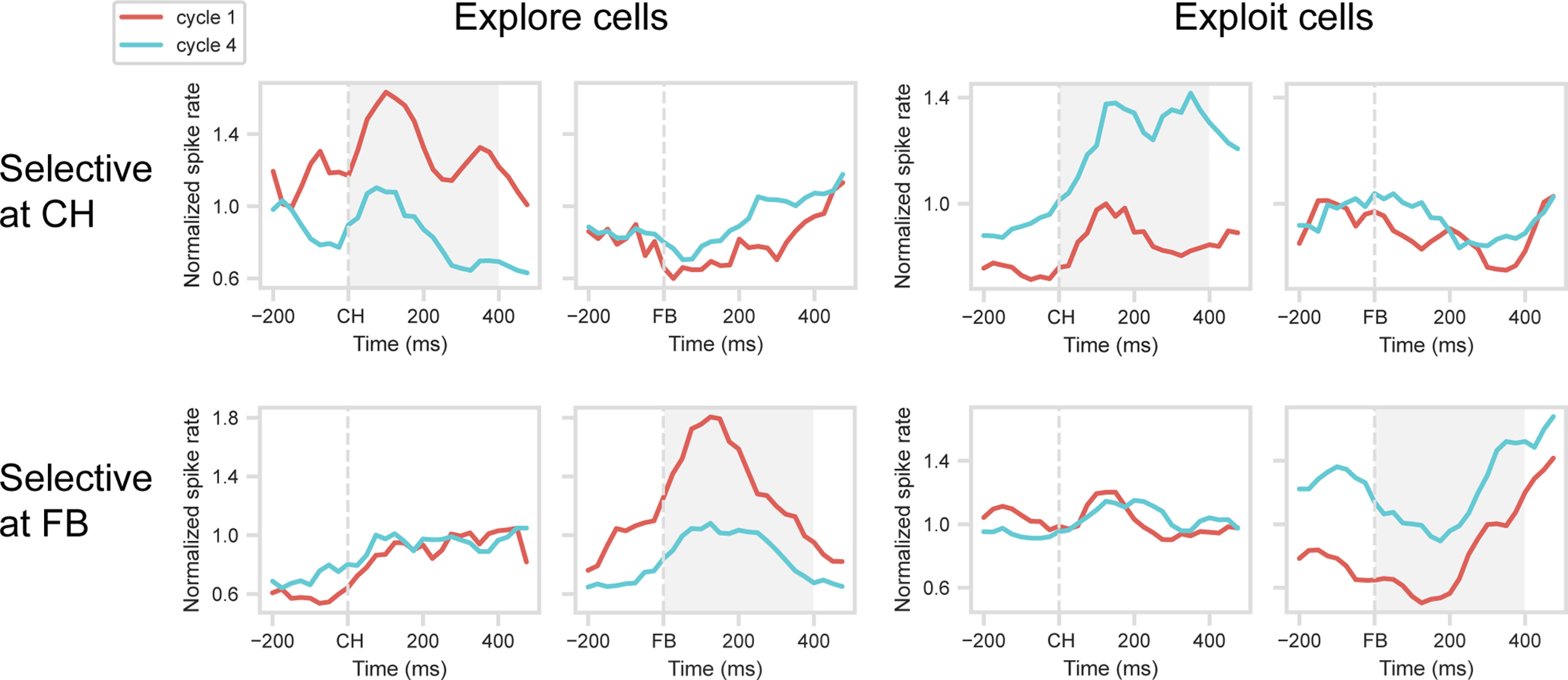
Validation of explore/exploit effect. Mean normalized spike rate for explore (spike rate Cycle 1 > Cycle 4) and exploit (spike rate Cycle 4 > Cycle 1) cells, identified at CH and FB periods. Data are cross-validated, with separate trials used to identify selective cells (selection dataset) and to construct PSTHs (validation dataset). Gray shading represents analysis windows (CH for CH-selected cells, FB for FB-selected cells).

In the selection dataset, for the CH period, the main effect of cycle was significant in 44 cells (17.3% of total): 18 with a preference for Cycle 1, 26 for Cycle 4. Mean PSTHs for these cells, calculated in the validation dataset, are shown in the upper row of Figure 3, with explore (Cycle 1 preferring) cells in the left panel, exploit (Cycle 4 preferring) cells on the right. *t* tests on the window 0-400 ms from CH onset confirmed significant cycle preferences in the validation data (for explore cells, *t*_(17)_ = 4.58, *p* < 0.001; for exploit cells, *t*_(25)_ = 4.39, *p* < 0.001). PSTHs suggest sustained cycle preferences that began even before CH onset. Notably, however, cells selected for a cycle preference during CH showed no evidence of a similar preference around FB.

A similar picture is evident for cells with cycle preference at FB (Fig. 3, bottom row). In the selection dataset, ANOVA on the FB period showed a significant main effect of cycle in 52 cells (20.5% of total): 15 with a preference for Cycle 1, 37 for Cycle 4. PSTHs for the validation dataset suggest sustained cycle preferences around FB, with *t* tests confirming significant cycle preferences in the 0-400 ms window (for explore cells, *t*_(14)_ = 2.94, *p* < 0.05; for exploit cells, *t*_(36)_ = 6.61, *p* < 0.001). Again, however, cells selected for a cycle preference during FB showed no evidence of a similar preference around CH.

Explore/exploit (Cycle 1/Cycle 4) preferences were largely stable across target objects. When ANOVAs on the selection dataset were repeated separately for each object set, now with an additional factor of object, only 9% of explore/exploit cells at CH, and 8% of explore/exploit cells at FB, showed a significant interaction (*p* < 0.05) between cycle and object (average results for the two stimulus sets).

These initial analyses show that substantial fractions of prefrontal cells differentiate the processes of explore and exploit. Although explore/exploit preferences are seen at both CH and FB, preferences at these two stages of a trial are unrelated, implying selectivity for the conjunction of cycle (explore/exploit) and trial stage (CH/FB).

### Temporal cross-generalization of cycle preferences

**Figure 4.**
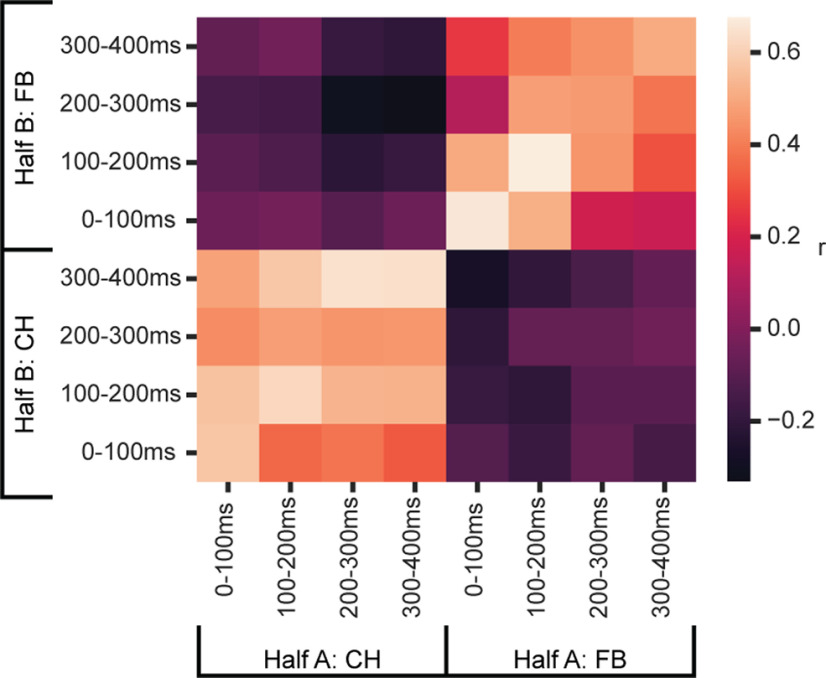
Temporal structure of explore/exploit preference. For each cell, trials were split into two groups (half A and B), and within each group of trials, cycle preference (Cycle 4 spike rate minus Cycle 1 spike rate) was calculated in 4 × 100 ms windows following onset of CH and FB. For each time window, this produced two independent vectors of 254 cycle preferences (one per cell). Data are correlations between half A and half B vectors.

To confirm that cycle preference is stable within a task phase (CH, FB) but not across task phases, we used a temporal cross-generalization analysis. We again randomly assigned trials for each cell to one of two groups (half A and half B; labels used in Fig. 4), and for each group of trials, subtracted mean activity in Cycle 1 from mean activity in Cycle 4. To remove effects of touched location (1-4) and number of targets (1, 2), we used unweighted means across these variables. For this analysis, we used 100 ms windows, four windows from onset of CH and four from onset of FB. For each window, this produced 2 vectors of 254 Cycle 4-1 differences, one for each half of the data, where 254 is the number of recorded cells. Correlations between vectors from the two halves of the data are shown in Figure 4. Strong correlations within CH and FB periods show that, within each period, the preference for Cycle 1 versus Cycle 4 was stable; between periods, however, correlations close to zero show unrelated cycle preferences.

### One-shot learning: single-cell responses

Having established that patterns of frontal activity differentiate explore and exploit, we moved on to examine the transition between these patterns with rapid learning. For this purpose, we focused on the one-shot learning seen in most 1-target problems. To mirror this rapid change in behavior, we searched for a similar one-shot change in the activity of explore/exploit cells. We focused on the explore and exploit cells identified above, and examined their detailed behavior during the rapid learning of 1-target problems. Again, these analyses used just the validation dataset from Cycles 1 and 4, along with all trials from Cycles 2 and 3.

**Figure 5.**
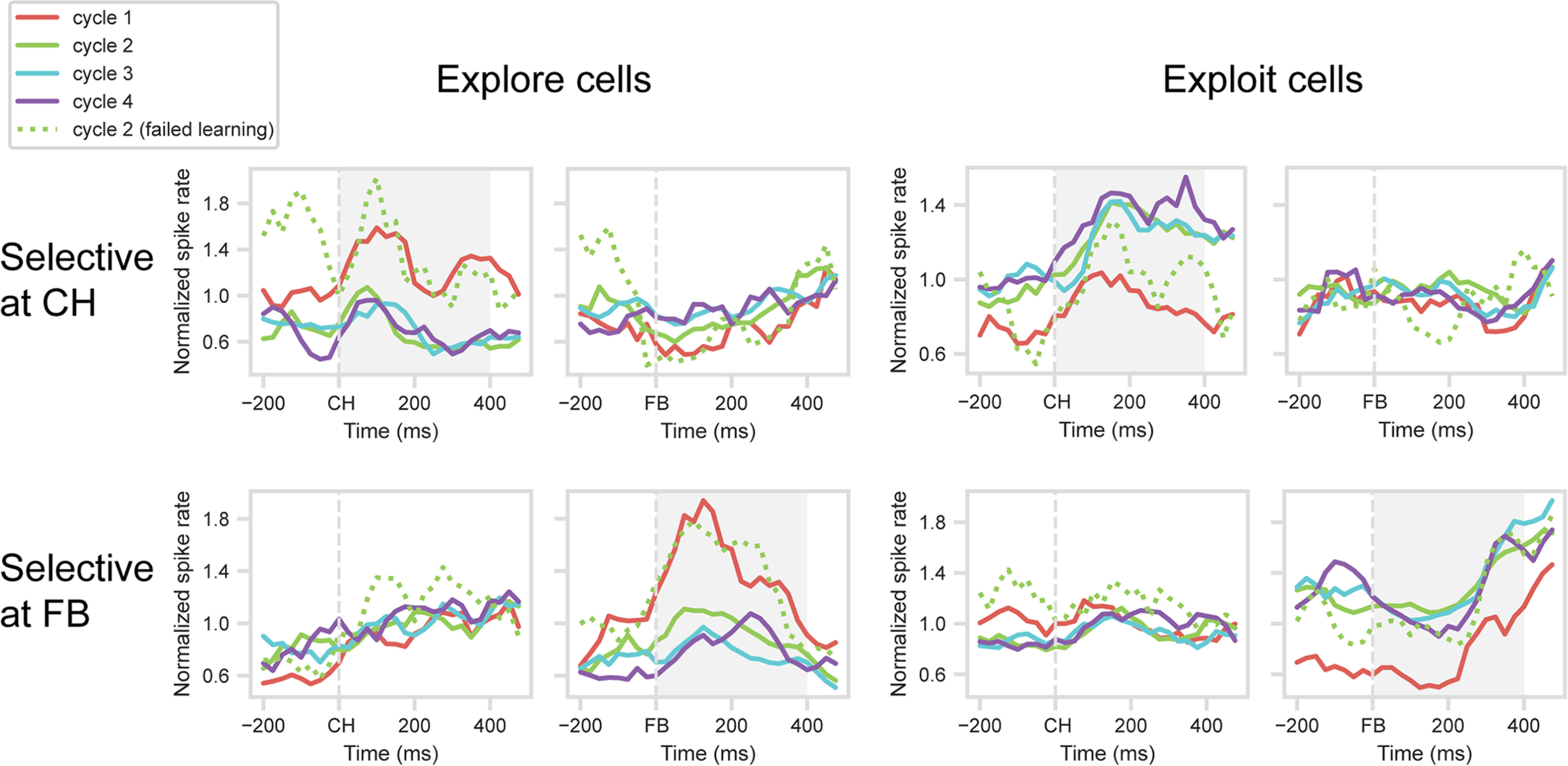
One-shot learning response of selected cells in PFC. One-target problems: mean normalized spike rates in each cycle. Cycle 1, all correct trials. Cycles 2-4, solid lines indicate correct trials, excluding cases with preceding error in this cycle. Cycle 2, dotted line indicates correct trials preceded by Cycle 2 error. Cell selection and cross-validation (Cycles 1 and 4 only) as for Figure 3. Gray shading represents analysis windows (CH for CH-selected cells, FB for FB-selected cells).

For the main analysis, we compared activity across Cycles 1-4. To focus on successful rapid learning, for Cycles 2-4 we excluded the exceptional cases in which the first response of the cycle was incorrect. Mean PSTHs for the same four groups of cells were calculated as before. Results are shown in the solid lines in Figure 5. Across the four cell groups, the results were clear-cut, with activity on Cycle 2 immediately switching from the Cycle 1 to the Cycle 4 pattern. For cells with an explore (Cycle 1) or exploit (Cycle 4) preference at CH (Fig. 5, top row), tests on the 400 ms window following CH onset showed significant differences between Cycle 1 and Cycle 2 (explore cells, *t*_(17)_ = 3.91, *p* = 0.001; exploit cells, *t*_(25)_ = 4.17, *p* = 0.001), but no significant differences between Cycles 2-4 (explore cells, *F*_(2,34)_ = 0.03; exploit cells, *F*_(2,50)_ = 1.08). For cells with an explore (Cycle 1) or exploit (Cycle 4) preference at FB (Fig. 5, bottom row), similar results were obtained in the 400 ms window following FB onset. The difference between Cycles 1 and 2 approached significance for explore cells (*t*_(14)_ = 2.06, *p* = 0.058) and was significant for exploit cells (*t*_(36)_ = 7.54, *p* < 0.001), while differences between Cycles 2-4 were not significant (explore cells, *F*_(2,28)_ = 2.26; exploit cells, *F*_(2,72)_ = 0.25).

In a supplementary analysis, we examined correct trials in Cycle 2 that followed at least one Cycle 2 error, a pattern suggesting failed learning on Cycle 1 and continued exploration in Cycle 2. For the two groups of explore cells, activity on these failed-learning trials resembled activity for Cycle 1 (Fig. 5, dotted green lines). Comparing failed-learning trials to regular Cycle 2 trials, where the first response was correct (Fig. 5, solid green lines), showed significant differences for both groups of cells (CH group, *t*_(17)_ = 3.66, *p* < 0.01; FB group, *t*_(14)_ = 3.19, *p* < 0.01). For exploit cells, results were less clear, with no significant difference between failed-learning and regular Cycle 2 trials (CH group, *t*_(25)_ = 1.57; FB group, *t*_(36)_ = 0.79), and for FB cells, a significant difference between failed-learning and Cycle 1 (*t*_(36)_ = 2.78, *p* < 0.01). These data show that, if learning was not complete after Cycle 1, the frontal explore state was partially preserved into Cycle 2.

### One-shot learning: population response

**Figure 6.**
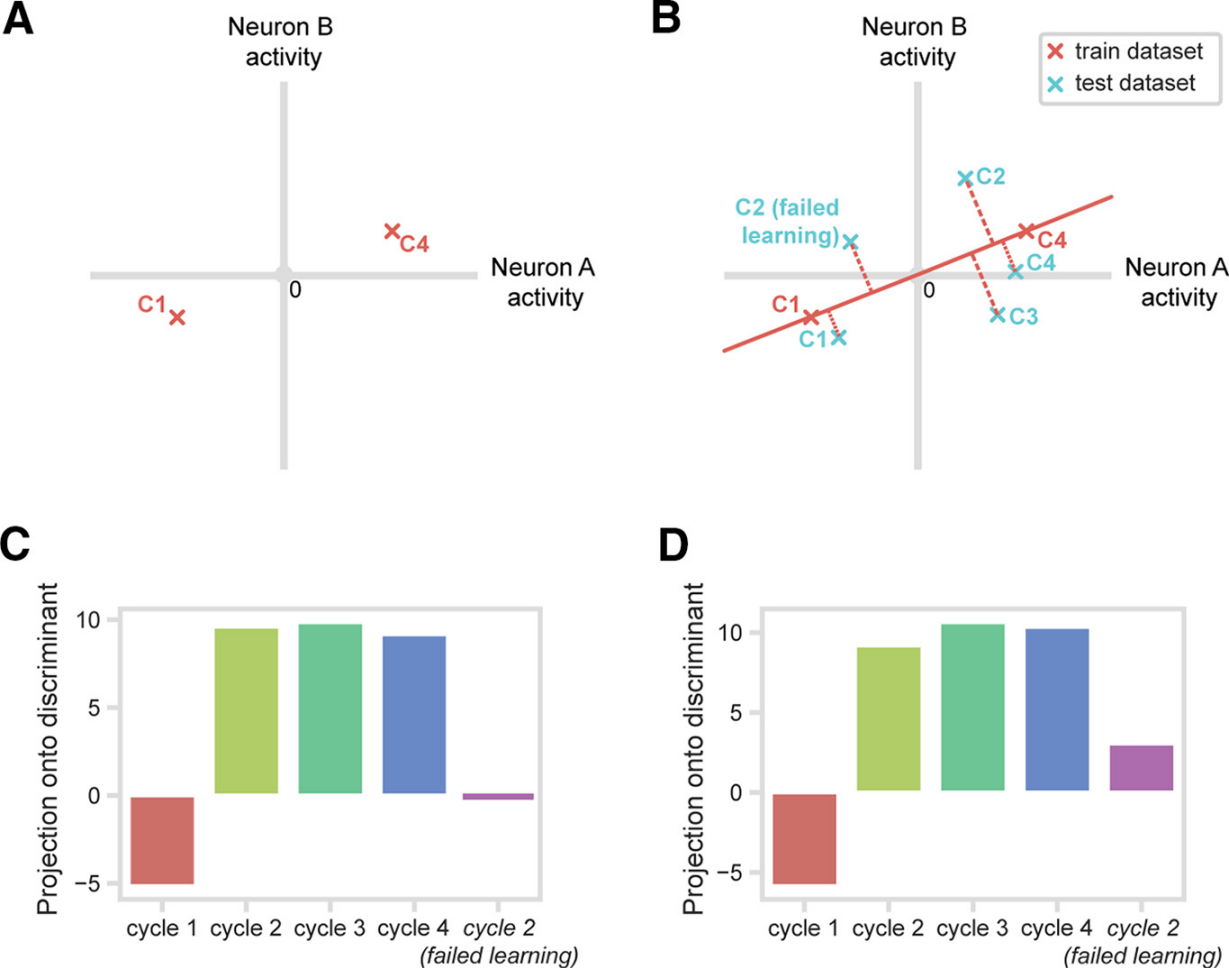
One-shot learning response of PFC population. ***A***, For each analysis window, a linear discriminant between population responses to Cycle 1 (C1) and Cycle 4 (C4) was calculated from a train dataset consisting of mean-centered, scaled firing rates for each neuron. The discriminant was calculated in the full 254-dimensional space based on firing rates of all neurons, shown schematically here for just two neurons. ***B***, Population activities from test data in Cycles 1 and 4 were projected onto the discriminant. The same procedure was followed for data from Cycle 2, Cycle 3, and Cycle 2 failed-learning trials. ***C***, Discriminant projections: CH window. First four bars, regular trials; fifth bar, Cycle 2, failed-learning trials. ***D***, Corresponding data for FB window.

Complementing the analysis of single neurons, we went on to examine the explore/exploit transition in the population activity of the entire cell sample of 254 PFC cells. Specifically, we aimed to quantify the similarity of population activity across the different cycles of learning. We constructed a linear discriminant separating population activity in Cycles 1 and 4 (Fig. 6*A*), then measured where activity in Cycles 2 and 3 (regular trials only, as above) fell on this discriminant (Fig. 6*B*). In a supplementary analysis, as before, we examined the failed-learning trials in Cycle 2 (i.e., correct trials following at least one Cycle 2 error).

Again, the analysis was conducted separately on activity in 400 ms windows following onset of CH and FB. For each window, as before, trials from Cycles 1 and 4 were randomly divided into two groups, with one group of trials used to construct the discriminant (train dataset), and the other to test it (test dataset). For each neuron, activity from the train dataset was expressed as deviation from this neuron's mean firing rate (unweighted mean, Cycles 1 and 4), scaled by the within-condition SD (unweighted mean of across-trial SDs, Cycles 1 and 4). Thus, activity in each cycle was described as a 254-dimensional vector of mean-centered, scaled firing rates, one per cell. We calculated the discriminant by simply subtracting the vector for Cycle 1 from the vector for Cycle 4. Test data from Cycles 1 and 4 were then mean-centered and scaled in the same way, using means and SDs from the train data, and projections onto the discriminant were obtained simply as the dot product between vectors for these scaled data and the discriminant. The same procedure was used for projections of data from Cycles 3 and 4, along with failed-learning trials from Cycle 2.

Results are shown in Figure 6*C* (CH) and Figure 6*D* (FB). As predicted, we found that Cycles 2-4 form one cluster independent of Cycle 1. Also matching results from the single-neuron analysis, failed-learning trials from Cycle 2 were closer to Cycle 1 than to regular Cycle 2 trials. For statistical testing, we used a permutation approach comparing projections for pairs of conditions. To compare Cycle 1 (test data) and Cycle 2, for example, for each neuron, we selected all Cycle 1 (test) and Cycle 2 trials, then randomized the cycle labels before calculating mean firing rates for these randomized data. This procedure was repeated for each neuron, projections on the original discriminant were recalculated, and the difference between projections for Cycle 1 and Cycle 2 was obtained. This whole procedure was repeated 1000 times, giving a null distribution against which the true data could be compared. The *p* value for the contrast was measured as the proportion of (absolute) distances in the permuted data greater than the value in the true data. In line with Figure 6*C*, *D*, projections for Cycles 2 and 3 were significantly different from the projection for Cycle 1 (*p* < 0.001 for each comparison in both CH and FB periods). Cycles 2 and 3 did not differ from Cycle 4 (*p* > 0.10 for all comparisons). For failed-learning trials in Cycle 2, projections differed significantly from both Cycle 1 (*p* < 0.001 for both CH and FB) and Cycle 2 (*p* < 0.001 for both CH and FB) regular trials.

### Expectancy and error

Slow, incremental learning is critically driven by reward prediction and prediction error. In the explore cycle of our task, expectancy of reward would increase over successive trials, while at feedback there would be variable positive and negative prediction errors. As these factors would be absent in exploit cycles, we wondered whether differences between explore and exploit cells might in part reflect differing sensitivity to prediction and error. To test for this, we used activity in Cycle 1, when the outcome of each selection was uncertain. Again, we focused on the explore and exploit cell groups defined above; and except where noted, trials from the selection dataset were removed from the analyses.

First, we asked how the activity of explore and exploit cells changed over the course of Cycle 1, as more objects were sampled and eliminated, and the expectancy of reward progressively increased. For 1-target problems, we sorted correct (rewarded) Cycle 1 trials according to whether the object selected was the first, second, third, or fourth different object sampled in this cycle. For this analysis, it was impossible to cross the factors of sample order and selected object location. On the first trial of Cycle 1, animals had very strong preferences for choosing the object in a particular location, which we call the animal's favored location. Strong preference for this location meant that, for this first trial, data were not always available for other locations. To ensure that results were not biased by location differences, we analyzed data just from trials in which the target was found in the animal's favored location, after sampling 0, 1, 2, or 3 other objects on previous trials of the cycle (in any locations; note random repositioning of objects on each trial).

**Figure 7.**
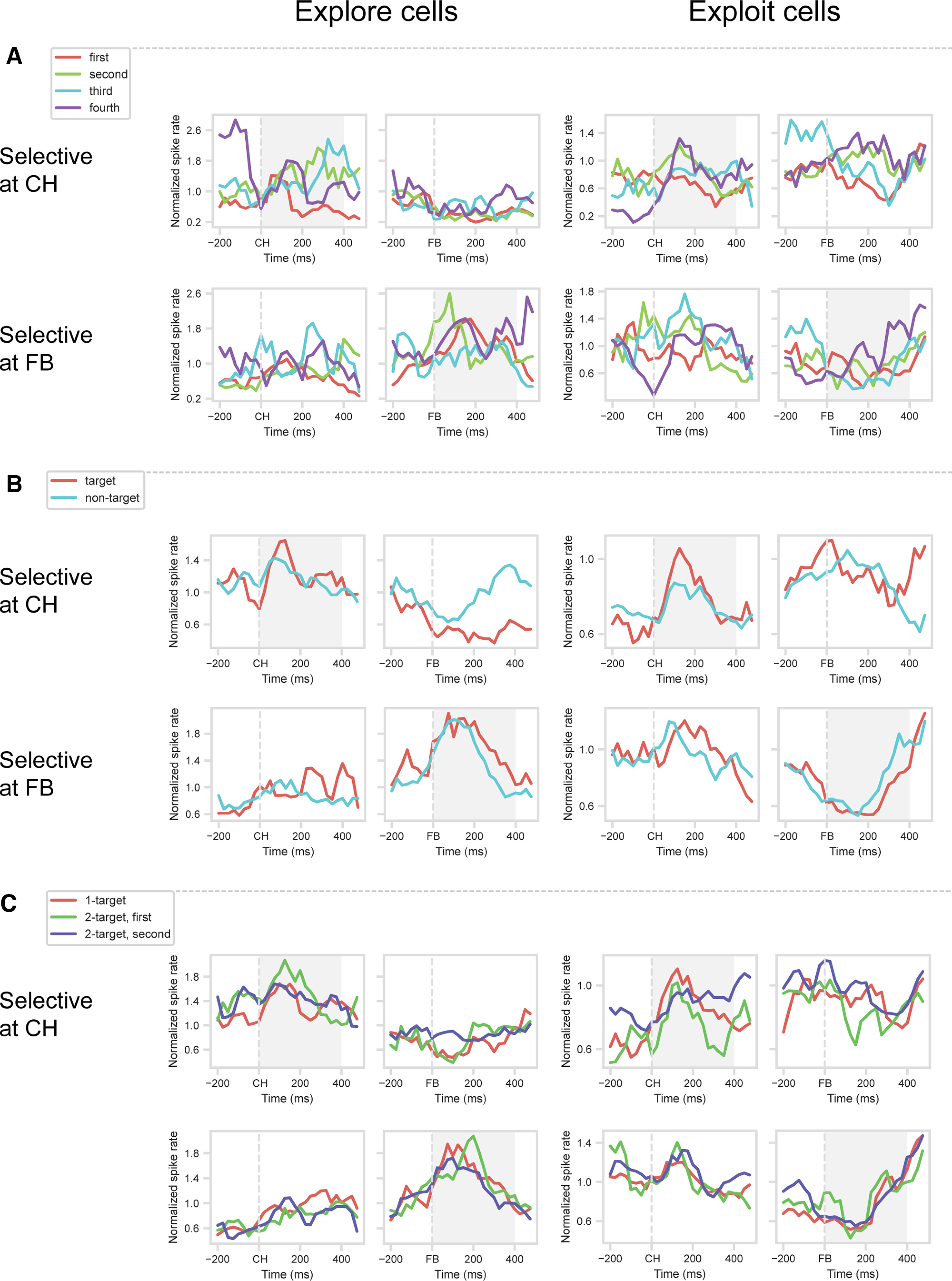
Expectancy and error. ***A–C***, Cell selection and windows as Figure 3. ***A***, One-target problems, Cycle 1: Target first, second, third, or fourth object sampled. Data just for targets in animal's favored location. ***B***, One-target problems, Cycle 1: Responses on correct (target selected) and incorrect (nontarget selected) trials. Data just for targets in animal's favored location, averaged across first, second, or third object sampled in cycle. ***C***, Cycle 1: Comparison of 1-target problems with first and second targets discovered in 2-target problems.

Inconsistent with a progressive increase in reward expectancy, the results (Fig. 7*A*) showed no significant effects of sampling order. For cells with an explore (Cycle 1) or exploit (Cycle 4) preference at CH (top row), ANOVAs on the 400 ms window following CH onset showed no significant effects of sampling order (explore cells, *F*_(3,45)_ = 2.21; exploit cells, *F*_(3,69)_ = 0.88). For cells with an explore (Cycle 1) or exploit (Cycle 4) preference at FB (bottom row), similar results were obtained in the 400 ms window following FB onset (explore cells, *F*_(3,36)_ = 0.97; exploit cells, *F*_(3,102)_ = 1.70). Thus, explore cells, as a group, did not show progressively decreasing activity with increasing reward expectancy, but rather a binary decrease from explore to exploit. Complementarily, exploit cells as a group did not show progressively increasing activity with increasing reward expectancy, but rather a binary increase from explore to exploit.

Second, to examine effects of prediction error, we compared activity on correct (target selection) and incorrect (nontarget selection) trials, again using Cycle 1 data from 1-target problems. To eliminate effects of serial position in the cycle, we ignored data for revisits, and compared correct and incorrect trials unweighted for serial position (average of responses separately calculated for the first, second, and third objects sampled in the cycle; incorrect impossible for object sampled fourth). To eliminate potential confounding effects of selected location, again we used only trials with selection of the animal's favored location. At both CH and FB, results suggested little mean difference between correct and incorrect trials (Fig. 7*B*). For cells with an explore (Cycle 1) or exploit (Cycle 4) preference at CH (top row), *t* tests on the 400 ms window following CH onset showed no significant differences between target and nontarget trials (explore cells, *t*_(17)_ = 0.71; exploit cells, *t*_(25)_ = 0.84). For cells with an explore (Cycle 1) or exploit (Cycle 4) preference at FB (bottom row), similar results were obtained in the 400 ms window following FB onset (explore cells, *t*_(14)_ = 1.33; exploit cells, *t*_(36)_ = 0.96). Thus, FB explore cells, as a group, did not show strong response to positive prediction error, but rather a binary decrease in activity from explore to exploit, while FB exploit cells, as a group, did not show reduced activity for positive prediction error.

In the cell sample as a whole, there was frequent discrimination of corrects and errors in the FB period. ANOVA with factors correct/error × object set × selected location, this time, including all trials (selection and validation datasets combined), showed that, in the whole sample of 254 cells, there were 58 (22.8%) with a main effect of correct/error: 24 preferring correct and 34 preferring error. Of the 52 explore/exploit cells defined in our main analysis at FB, 16 (30.8%) also showed a significant difference between corrects and errors. Thus, outcome information was encoded in prefrontal cells, but neither explore nor exploit cells consistently favored positive or negative outcome.

Finally, we compared target-discovery trials in 1-target problems with first and second targets discovered in 2-target problems. Again, these cases have very different reward expectancies; for example, in a 1-target problem, the first object selected has only a 0.25 probability of being a target, whereas for a 2-target problem, this probability is 0.5. Again, however, results showed very similar responses for these three types of Cycle 1 target trials (Fig. 7*C*). For cells with an explore (Cycle 1) or exploit (Cycle 4) preference at CH (top row), ANOVAs on the 400 ms window following CH onset showed no significant differences between the three trial types (explore cells, *F*_(2,34)_ = 0.21; exploit cells, *F*_(2,50)_ = 2.78). For cells with an explore (Cycle 1) or exploit (Cycle 4) preference at FB (bottom row), similar results were obtained in the 400 ms window following FB onset (explore cells, *F*_(2,28)_ = 0.37; exploit cells, *F*_(2,72)_ = 0.25).

Contrary to incremental changes in reward prediction, these data show that explore/exploit selectivity was approximately binary, distinguishing simply an explore state, in which new information was sought, and an exploit state, in which known information was used.

### Parietal activity

**Figure 8.**
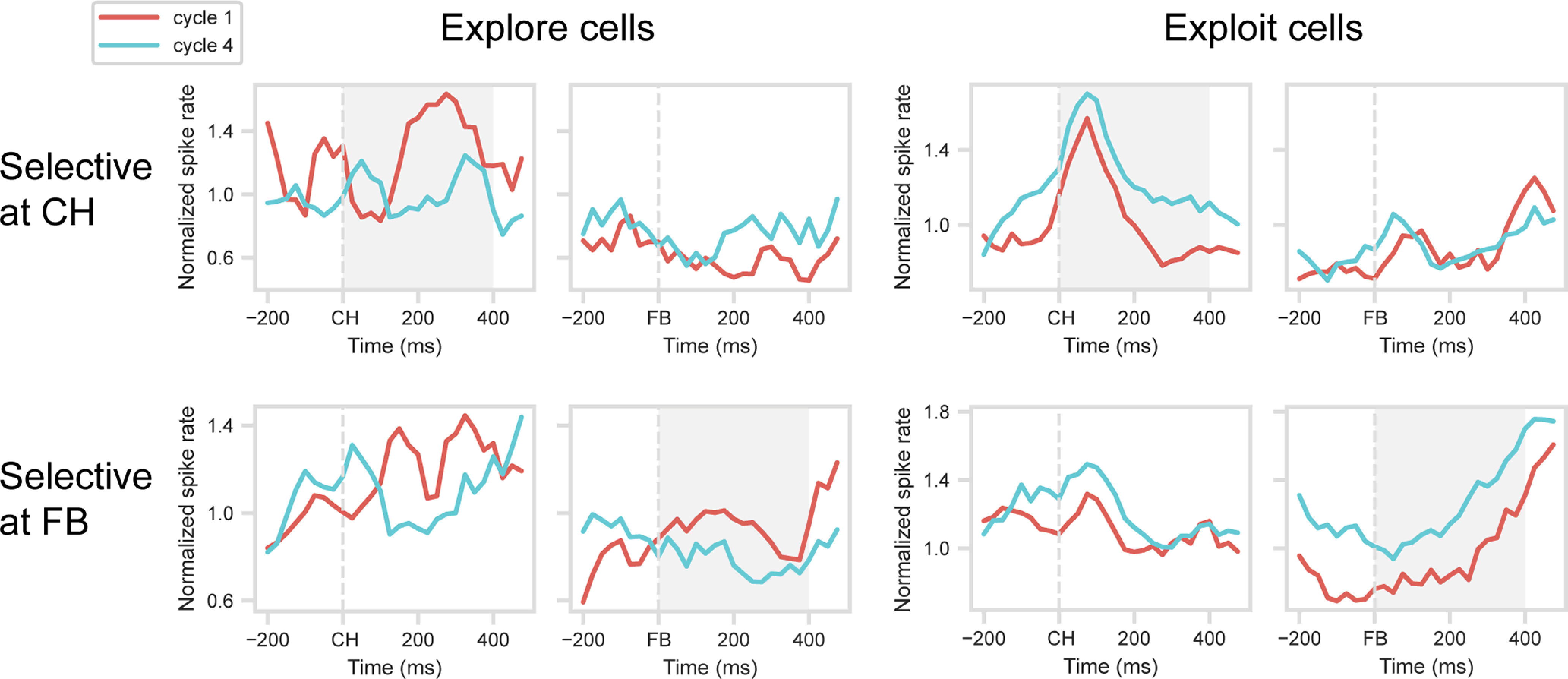
Explore/exploit preference in inferior parietal cortex. Layout as in Figure 3.

Finally, parallel analyses were conducted on a population of 170 cells recorded in inferior parietal cortex (for details, see Kadohisa et al., 2020). For the CH period, the difference in activity between Cycles 1 and 4, tested as before on a selection dataset, was significant in 20 cells (11.8% of total): 5 with a preference for Cycle 1, 15 for Cycle 4. For the FB period, the difference was significant in 32 cells (18.8%): 10 with a preference for Cycle 1, 22 for Cycle 4. In this case, testing with a validation dataset produced mixed results, although trends in the data (Fig. 8) weakly resembled those found in frontal cells. For explore (Cycle 1 preference) or exploit (Cycle 4 preference) cells identified at CH (top row), *t* tests on the 400 ms window following CH onset showed no significant difference between cycles for explore cells (*t*_(4)_ = 0.80) but a significant difference for exploit cells (*t*_(14)_ = 3.54, *p* < 0.001). For cells with an explore (Cycle 1) or exploit (Cycle 4) preference at FB (bottom row), similarly mixed results were obtained in the 400 ms window following FB onset (explore cells, *t*_(9)_ = 1.15; exploit cells, *t*_(21)_ = 3.65, *p* < 0.01). Although these cross-validated results were weak, direct comparisons with frontal results (one ANOVA corresponding to each of the above *t* tests, factors region × cycle) showed no reliable differences between regions (region × cycle interaction, all *F* < 2.2).

## Discussion

While learning can be slow when being done from scratch, well-developed internal task models may be generalized to a newly encountered situation. In this case, new stimuli can be quickly bound to their roles within the task model. We examined the transition from novel information seeking to known information use within a well-learned task structure.

In each new problem, monkeys learned which target objects were rewarded when touched. At the explore (information seeking) stage, objects were sampled over a series of trials until rewards were obtained. In subsequent exploit cycles, the structure of each trial was similar, but now the monkey could base choices on previously acquired information. The monkey's behavior, though not perfect, showed a clear model of task rules, with few selections of previously sampled nontargets, and in 2-target problems, few selections of a target already chosen in the current cycle. For the easier, 1-target problems, learning was typically one-shot, with just a single reward in Cycle 1 sufficient to produce consistent target selection.

Our results showed different patterns of prefrontal activity for explore and exploit stages of each problem. For many cells, activity differentiated Cycle 1, when new information was acquired, from later cycles when this information was used to control subsequent behavior. In 1-target problems, corresponding to one-shot learning in behavior, the switch in frontal activity was close to binary, with little subsequent change after the first exploit trial. When learning was not complete after the first success trial, furthermore, the data showed partial preservation of the frontal explore state. These results held both at the level of single cells with explore/exploit preferences, and at the level of activity pattern across the whole-cell population.

Learning within a known task structure calls for different computations during initial binding of stimuli to their roles (novel information seeking), and subsequent retrieval and use of these bindings (known information use). For example, in deep reinforcement learning, meta-learning of a task model endows agents with the ability to quickly adapt their behavior to match a new reward structure in an otherwise known environment (Botvinick et al., 2020). Modeling studies have examined how progressive learning in neural networks can shape connectivity to implement required cognitive operations (Barak et al., 2013; Chaisangmongkon et al., 2017). In an important set of simulations Wang et al. (2018) show how agents can quickly switch from learning to retrieval of novel variable bindings during prelearned task phases. Our data show that PFC produces a discrete code for these different task phases.

Many signals associated with learning have previously been studied. Neurons in various regions, including lateral frontal cortex, selectively code for errors (Gehring and Knight, 2000; Mansouri et al., 2006), the effects of expectations (Matsumoto et al., 2003; Rouault et al., 2019), decision confidence (De Martino et al., 2013; Boldt and Yeung, 2015), and reward prediction errors (Tanaka et al., 2004; Kennerley et al., 2011). Consistent with the hypothesis that those are an integral part of many learning processes, we found many prefrontal cells coding for errors. Preference for correct versus error trials, however, was rather independent of explore/exploit preferences. Similarly, neither explore nor exploit cells showed progressive changes in activity with changing reward expectancy. This relative independence of cycle preference from outcome and expectancy coding suggests that PFC constructs an additional binary code to support the rapid explore/exploit switch.

Rapid learning will always require an agent to have well-tuned priors built up through experience with the environment at hand. Before starting recording sessions, monkeys received extensive training. During this learning, monkeys familiarized themselves not only with the task structure but also the different object sets, and hence built up very narrow priors to support rapid learning. We thus studied a highly contextualized form of learning, where monkeys only need to learn which known stimulus to pair with a known process in the task (i.e., touch target object d). In other tasks, for example when subjects need to pair an unknown stimulus to a known task process (Harlow, 1949; Cook and Fagot, 2009) or pair an unknown stimulus to an unknown task process (Youssef-Shalala et al., 2014; Franklin and Frank, 2020), broader priors/more general “meta task models” will be needed to support learning. PFC might use the most rapid shift from explore to exploit when simply generalizing over stimuli, but fall back onto more classical continuous learning signals with more substantial change in task conditions (Wilson et al., 2014; Wu et al., 2018).

Although exploit trials involved repeated selection of the same target object, the binary switch from explore to exploit is not well described as a simple repetition effect. Beyond Cycle 2, neither explore nor exploit cells showed further changes with additional choice repetitions. The partially preserved explore state following failed-learning trials in Cycle 1 also tells against a simple repetition account.

In PFC, activity patterns for different stages of a trial can be approximately orthogonal (Sigala et al., 2008; Kadohisa et al., 2020). Orthogonal patterns may minimize interference between the cognitive operations of successive task steps (Sigala et al., 2008). In the present data, preferences for explore versus exploit were independent during CH and FB. Internal models for these two stages of the task would involve different cognitive operations: for explore, novel choice generation at CH and new learning at FB, but for exploit, retrieval of the previously rewarded target at CH, and confirming a predicted success at FB. Conjunctive coding for combinations of trial phase (CH, FB) and knowledge state (explore, exploit) may be required to construct and direct the multiple stages of the abstract task model (Enel et al., 2016). These results match many reports of mixed selectivity in prefrontal cells (Mushiake et al., 2006; Rigotti et al., 2010, 2013; Warden and Miller, 2010).

In contrast with independent explore/exploit preferences at CH and FB, cross-temporal generalization showed rather stable preferences within each of these trial phases (Fig. 4). Although average PSTHs suggested some variation within each phase (Fig. 3), additional data would be needed to examine more fine-grained temporal structure.

Prior studies examined the explore to exploit transition in a spatial selection task (Procyk and Goldman-Rakic, 2006; Quilodran et al., 2008; Khamassi et al., 2015). Our results extend these findings in object selection task. In this task, we provide a detailed characterization of the explore to exploit transition, including one-shot switching, partial preservation of the explore state after a failed learning trial, independent explore/exploit coding at choice and feedback stages of the trial, and temporal stability within each of these stages.

If frontal activity binds objects to their roles in the task, there must be a representation of object identity. In tasks like ours, sustained firing frontal patterns can carry important information in working memory (Fuster and Alexander, 1971; Funahashi et al., 1989; Constantinidis et al., 2018). When targets were discovered in Cycle 1, sustained object-selective activity could have carried this target information through to later cycles. Our previous analyses of object selectivity, however, show that this does not happen in our task (Kadohisa et al., 2020). Although frontal neurons code object information at FB and CH, these two codes are orthogonal, and between trials, all object information disappears. As successive task operations take place, object information is newly implemented in the pattern of frontal activity.

Many studies show closely similar neural properties in lateral prefrontal and inferior parietal cortex (Chafee and Goldman-Rakic, 1998; Goodwin et al., 2012; Brincat et al., 2018; Meyers et al., 2018). In the current task, prefrontal and inferior parietal neurons show similar coding of target identity and location (Kadohisa et al., 2020). The data suggest that, like prefrontal cells, inferior parietal cells can also show explore/exploit preferences, although only for some cell groups was the difference significant in the cross-validated test.

Previous findings from both human imaging (Konishi et al., 1998; Hampshire and Owen, 2006) and single-cell physiology (Procyk and Goldman-Rakic, 2006; Quilodran et al., 2008) suggest a reduction in frontal activity with the transition from unknown to known task rules, or more broadly over the early trials of a new task (Ruge et al., 2019). In contrast to this simple change, we observe cells with both increased and decreased activity with the switch from explore to exploit. Both explore and exploit preferences may be important to direct the different cognitive operations of constructing and using the task model.

In one-shot learning, newly acquired information is bound to its role within a previously learned, abstract task model. Building on previous findings (Procyk and Goldman-Rakic, 2006; Quilodran et al., 2008), our data show a one-shot switch of firing rate in many prefrontal cells, matching one-shot behavioral learning. This switch of neural activity occurs independently at different stages of a trial, with their different cognitive requirements. The binary switch in frontal activity may enable one-shot switch between cognitive operations of information seeking and information use. More generally, such switches may allow the high-speed adaptability that characterizes much animal and human behavior.
